# Advances in the treatment of acute graft-versus-host disease

**DOI:** 10.1111/jcmm.12093

**Published:** 2013-06-26

**Authors:** Liren Qian, Zhengcheng Wu, Jianliang Shen

**Affiliations:** a Department of Haematology, Navy General HospitalBeijing, China; b Department of Neurology, Navy General HospitalBeijing, China

**Keywords:** Acute graft-versus-host disease, stem cell transplantation, hydrogen, treatment

## Abstract

Allogeneic hematopoietic stem cell transplantation (HSCT) has been widely used for the treatment of hematologic malignant and non-malignant hematologic diseases and other diseases. However, acute graft-versus-host disease (GVHD) is a life-threatening complication of allogeneic transplantation. Acute GVHD may occur in 30% of transplant recipients, which is a syndrome of erythematous skin eruption, cholestatic liver disease and intestinal dysfunction, resulting from the activation of donor T lymphocytes by host antigen-presenting cells, resulting in an immune-mediated inflammatory response. Recent scientific advances in the understanding of the pathogenesis involved in the development of acute GVHD and clinical investigation have provided more effective therapeutic strategies for acute GVHD. This review focuses on major scientific and clinical advances in the treatment of acute GVHD.

- Introduction
- Acute Graft-versus-host disease (GVHD)
- Pathophysiology of acute GVHD
- Prevention of acute GVHD
- Treatment of acute GVHD
- First-line therapy
- A recent phase II trial conducted by the blood and marrow transplant
- Second-line therapy
- Future directions

## Introduction

Hematopoietic stem cell transplantation (HSCT) has been widely used for the treatment of hematologic malignant and non-malignant hematologic diseases and other diseases (Table 1). However, the widespread application of HSCT is restricted by the poor availability of suitable donors [1–4]. Acute graft-versus-host disease (GVHD) remains a major cause of post-transplant morbidity and mortality, even in patients who receive human leucocyte antigen (HLA) identical sibling grafts [5, 6]. Even through use of adequate post-transplantation immunosuppressive therapy, successfully engrafted recipients, free from active GVHD, immune response often show delayed immune reconstitution and remain susceptible to fatal infection [5–8]. Thus, acute GVHD continues to be a major limitation to successful HSCT. The objective of this review was to offer an overview of current management of acute GVHD regarding the pathophysiology, regimens in common clinical use, and regimens under investigation.

**Table 1:** List of diseases treated by hematopoietic stem cell transplantation [145]–[150]

Malignant disease
Acute myelogenous leukaemia
Acute lymphoblastic leukaemia
Chronic myelogenous leukaemia
Chronic lymphocytic leukaemia
Non-Hodgkin lymphoma
Hodgkin lymphoma
Multiple Myeloma
Myelodysplastic syndromes
Myeloproliferative syndromes
Waldenstrom macroglobulinemia
Hairy cell leukaemia
Amyloidosis
Testicular cancer
Paediatric tumorus
Neuroblastoma
Nonmalignant diseases
Acquired aplastic anaemia
Diamond-Blackfan syndrome
Dyskeratosis congenita/Hoyeraal-Hreidarsson syndrome
Fanconi anaemia
Shwachman-Diamond syndrome
Thalassemia
Sickle cell disease
Paroxysmal nocturnal hemoglobinuria
Severe combined immunodeficiency
Congenital leucocyte dysfunction
Osteopetrosis
Familial erythrophagocytic lymphohistiocytosis
Glanzmann disease
Hereditary storage diseases
Autoimmune lymphoproliferative syndrome (ALPS)
Ataxia-telangiectasia
Chediak-Higashi syndrome
Chronic granulomatous disease
Complete interferon-γ receptor 1 deficiency
DiGeorge syndrome
Familial hemophagocytic lymphohistiocytosis
Griscelli's syndrome
Hyper-IgM syndrome
Kostmann syndrome
Leucocyte adhesion deficiency
Wiskott-Aldrich syndrome
X-linked syndrome
Fucosidosis
Gaucher's disease
Mucopolysaccharidoses
Congenital erythropoietic porphyria (Günther's disease)
Essential thrombocytopenia
Histiocytoses
Idiopathic hypereosinophilic syndrome
Myelofibrosis
Polycythemia vera

## Acute Graft-versus-host disease

Acute GVHD remains a clinical challenge and a major source of morbidity and mortality following allogeneic HSCT. Traditionally, GVHD was divided into acute and chronic GVHD based on the timing of the onset of GVHD symptoms. Graft-versus-host disease occurs on or before the 100th day following transplantation was defined as acute GVHD, and the onset of chronic GVHD occurs after the 100th day. However, this temporal distinction is somewhat arbitrary, as patients may manifest classic signs of acute GVHD even after day 100, and chronic manifestations may occur before 100 days post-transplantation. Acute GVHD diagnosis should be confirmed by biopsy of an affected organ if possible; in addition, other non-GVHD complications involving the skin, liver and GI tract should be ruled out, such as cytomegalovirus enteritis or drug eruption from medications [9]. However, ultimate diagnosis of acute GVHD needs integration of all available clinical information, because the sensitivity of these biopsies is only approximately 60% [10]. Thus, the development of diagnostic tests for acute GVHD is needed [11]. Because long-term survival from acute GVHD is directly related to the severity of skin, liver and gut involvement, to facilitate the study and prognostication of acute GVHD, a clinical staging and grading system was developed. The severity score was clinically based and ranged between grades 0 and IV according to the Keystone 1994 consensus criteria, as defined by the involvement of each organ system (Table 2) [12]. The staging and grading system of acute GVHD has been updated by the Center for international Bone Marrow Transplant Registry (IBMTR) (IBMTR) criteria [13–15], in which acute GVHD can be diagnosed after 100 days post-transplantation, and patients manifest the only clinic signs including anorexia, nausea and vomiting with a positive upper gastrointestinal tract biopsy for acute GVHD are included under overall grade II acute GVHD.

**Table 2:** Staging and grading of acute graft-versus-host [12]

Stage	Skin	Liver	Gut
1	Rash <25% of body surface area	Bilirubin 2–3 mg/dl	Diarrhoea 500–1000 ml/day or persistent nausea
2	Rash 25–50% of body surface area	Bilirubin 3–6 mg/dl	Diarrhoea 1000–1500 ml/day
3	Rash >50% of body surface area	Bilirubin 6–15 mg/dl	Diarrhoea >1500 ml/day
4	Erythroderma with bullae formation	Bilirubin >15 mg/dl	Severe abdominal pain with or without ileus
Grade			
I	Stage 1–2	None	None
II	Stage 3 or	Stage 1 or	Stage 1
III		Stage 2–3 or	Stage 2–4
IV	Stage 4 or	Stage 4	

## Pathophysiology of acute GVHD

**Fig. 1:**
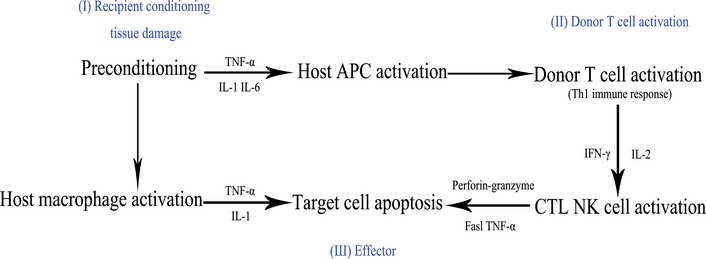
Pathophysiology of acute GVHD.

The pathophysiology of acute GVHD involves complex three stages as proposed by Ferrara and Reddy [16]. Stage I involves tissue damage and cellular activation induced by preconditioning (Fig. 1). The first phase occurs prior to transplantation of the graft, during which time the transplant conditioning regimen such as chemotherapy and irradiation, damages and activates host tissues leading to secretion of inflammatory cytokines [tumour necrosis factor-α (TNF-α), interleukin-1 (IL-1), IL-6 and IFN-γ], and danger signals such as adenosine-5′-triphosphate (ATP) and nicotine adenine dinucleotide, as well as extracellular matrix proteins such as biglycan that promote activation and maturation of antigen-presenting cells (APCs) [17–20].

Cytokine cascade plays an important role in the occurrence and severity of acute GVHD, and the polymorphism of cytokine genes have been shown to affect the severity of acute GVHD [21]. Stage II involves the activation of donor lymphocytes (T cells). Resting donor T cells become activated in secondary lymphoid organs by both recipient and donor APCs as well as inflammatory cytokines, which expand and differentiate into effector cells [18, 22]. Activated T cells result in the production of IL-2 and IFN-γ (Th1) [23] or secreting IL-4, IL-5, IL-10, and IL-13 (Th2). Interleukin-2 plays a central role in controlling and amplifying the allogeneic immune response [23, 24], activating further T cell and natural killer cell responses, priming macrophages to release TNF-α and further inflammation damages the skin, liver, and gut. Both IL-2 and its receptor have been and are used as targets for the management of acute GVHD [25–27].

In the third stage, cellular and inflammatory factors are released, including TNF-α, IL-1, IL-6, IL-10, IL-12, which underlie the clinical manifestations of acute GVHD [28, 29]. A polymorphism in the TNF-α gene was demonstrated to increase incidence of severe acute GHVD [30], but polymorphisms in IL-10, which was considered as suppressing TNF-α, IL-1, and other inflammatory cytokines, was demonstrated with the ability to decrease incidence of acute GVHD [31].

## Prevention of acute GVHD

The original prevention of acute GVHD developed since 1950s. In 1958, methotrexate (MTX) was used by Uphoff *et al*. because of its ability to delete proliferating donor lymphocytes through inhibition of dihydrofolate reductase and production of thymidylate and purines. In the1970s, cyclosporine was successfully used in the prevention of acute GVHD, which showed equivalency with MTX in prospective clinical studies by inhibiting T cell proliferation [32]. Since 1980s, calcineurin inhibitor cyclosporine and tacrolimus (TAC) combination with MTX have been successfully used in clinical trials, which laid the foundation of the following development of acute GVHD prophylaxis [32, 33]. In the 1990s, tacrolimus was used in a controlled clinical trial by Hiraoka *et al*. [34]. The incidence of grade II to IV acute GVHD within 100 days of transplantation was significantly lower among patients that received tacrolimus compared to patients that received cyclosporine. In two large randomized phase trials, TAC was used in combination with short-course MTX. Both trials showed reductions in overall acute GVHD incidence among patients receiving TAC/MTX. It has been demonstrated that TAC/MTX was superior to cyclosporine/MTX in the prevention of acute GVHD. Grade II-IV acute GVHD was significantly lower with TAC/MTX compared to cyclosporine/MTX both sibling donor (32% *versus* 44%; *P* = 0.01) and unrelated donor (56% *versus* 74%; *P* = 0.0002) transplant trials [35]. But a randomized trial comparing combination of cyclosporine and MTX to the combination of cyclosporine, prednisone, and MTX did not show any significant difference in acute GVHD incidence, relapse risk, and overall survival [8]. Nowadays, the combination of (cyclosporine or TAC) and MTX is the commonly used standard to prevent acute GVHD, which has been demonstrated better than single-agent MTX. [33, 36, 37] In recent clinical trials, post-transplant cyclophosphamide also promoted graft-host tolerance shows promise [38]. However, these agents have numerous side effects, including anorexia, nausea, vomiting and gastrointestinal tract reaction, gingival hyperplasia, renal toxicity, delayed cell count and immunological recovery, thrombotic microangiopathy, and posterior reversible encephalopathy syndrome, *et al*. [39–41] These side effects associated with methotrexate in particular has led investigators to examine the activity of alternative agents, such as tacrolimus combined with either mycophenolate mofetil (MMF) or sirolimus [42–46].

Mycophenolate mofetil inhibits proliferation of T lymphocytes *via* its metabolite mycophenolic acid and is a selective inhibitor of inosine monophosphate dehydrogenase, an enzyme critical to the de-novo synthesis of guanosine nucleotide, is now commonly used in combination with a calcineurin inhibitor for acute GVHD prophylaxis in preventing acute GVHD [47]. Mycophenolate mofetil and calcineurin inhibitor did not show better effect in acute GVHD prevention than MTX and calcineurin inhibitor, but the incidence and severity or oropharyngeal mucositis with the use of MMF was significantly decreased [42, 43, 48–52].

Approaches for the prevention of acute GVHD have utilized donor T cell depletion from the graft prior to infusion since 1980s [1, 53–55], by using physical techniques, density gradient centrifugation, anti-thymocyte globulin [56–59] or monoclonal antibody-based depletion methods, and CD34-cell-positive selection, *et al*. However, this approach is associated with a higher risk of graft rejection, impaired immune reconstitution, infectious complications, and relapse, and increased risk of primary disease relapse after HCT [60, 61]. Recent single-arm trials have shown 3-year disease-free survival approaching 60% with T cell-depleted peripheral blood stem cell transplantation [62, 63]. But T cell depletion did not improve overall survival, and the concept of partial marrow T cell depletion was evaluated by counterflow elutriation and T10B9 antibody plus complement in a multicenter randomized trial of 405 transplant recipients of HLA-matched unrelated donor grafts [64]. The incidence of acute GVHD grades II to IV was significantly lower in the partial T cell depletion group. However, partial marrow T cell depletion did not improve event-free and overall survival either. Besides, administration of the anti-CD52 antibody alemtuzumab (Campath 1H) was another approach to establish partial T cell depletion of the donor graft which was demonstrated could facilitate transplants from HLA-mismatched haploidentical donors without significant acute GVHD [65, 66]. This shows the promise of HSCT to patients without HLA-matched donors. But patients who received partial T cell depletion with administration of the anti-CD52 antibody were at increased risk for opportunistic infections, graft loss, and relapse. Therefore, investigators refined protocols for T cell depletion. In a phase II trial, Jakubowski *et al*. [63] reported *ex vivo* T cell depletion employing CD34 enrichment in 35 unrelated donor transplants. With no pharmacologic prophylaxis, acute GVHD grade II-III developed in 9% and chronic GVHD in 29% of patients. Fatal infections occurred in 5 of 35 (14%) patients. There was one late graft failure. The efficacy of this protocol was also confirmed by Devine *et al*. [62] in HLA-matched sibling donor transplantation. These results demonstrate that partial depletion of donor T cells provides protection against acute GVHD.

Agents attempt to block the cytokine pathways in the development of acute GVHD. In a recent phase 1/2 study, exciting new success has been reported in acute GVHD prevention using a well-tolerated oral CCR5 antagonist [67]. A strikingly low incidence of gastrointestinal and liver acute GVHD was observed in the study by using maraviroc which blocks T cell chemotaxis [67]. Interleukin-1 and TNF-α also play a central role in the development of acute GVHD, but drugs that target these cytokine/chemokine-receptor interactions (etanercept, infliximab) failed to improve incidence rates of acute GVHD [68, 69].

Other Agents are in the early stages of clinical development for acute GVHD prevention and give more promise [70]. Bortezomib, a proteasome inhibitor also show promise in acute GVHD prevention in a phase 1/2 study [71]. In experimental models of mismatched HSCT, T-regulatory cells (Tregs) suppressed lethal GVHD [72] and favoured post-transplantation immune reconstitution when co-infused with conventional T cells (Tcons) [73]. In humans, Tregs was also demonstrated preventing acute GVHD [74, 75] and promote immune reconstitution in HLA-haploidentical transplantation [74].

## Treatment of acute GVHD

### First-line therapy

The therapy of grade I acute GVHD should include topical therapy (topical steroid creams or topical tacrolimus) without the need for additional systemic immunosuppression. Suitable strengths of topical steroids are critically reviewed and detailed by Dignan *et al*. [76]. Adults should commence on 0.1% tacrolimus until resolution.

Glucocorticoids are the initial standard for treatment of grade II–IV acute GVHD, including methylprednisolone or prednisone at a dose of 1–2 mg/kg per day with subsequent gradual dose reduction once disease activity resolves [2, 77–79]. The optimal rate of tapering steroid doses after initial treatment has not been defined. Long–term prednisone therapy showed no advantage in a prospective randomized trial [80]. In general, steroids doses should be gradual reduced when acute GVHD manifestations start showing major improvement. Inappropriately rapid taper rates carry a risk of acute GVHD exacerbation or recurrence, whereas inappropriately slow taper rates increase the risk of steroid-related complications. Doses should be gradual reduced 0.2 mg/kg/day every 3–5 days, slower after prednisone doses are decreased to less than 20–30 mg/day [81]. The taper schedules provided in national, multicenter trials for acute GVHD, such as Blood and Marrow Transplant Clinical Trials Network (BMT CTN) 0302 or 0802, reflect current practice and are appropriate. The mechanism underline their effects may because of lympholytic effects and anti-inflammatory properties [82]. Higher doses of methylprednisolone (10 mg/kg/day) do not prevent evolution to grade III or IV acute GVHD or improve survival [77]. Mielcarek *et al*. reported that initial therapy of acute GVHD with low-dose prednisone (1.0 mg/kg) does not compromise patient outcome compared to standard dose prednisone (2 mg/kg/day) in a retrospective study in those with grade I/II disease. Definitive conclusions could not be drawn for those patients with grade III or IV disease because of the small numbers in this group [83]. Adverse effects of glucocorticoids include hypertension hyperglycemia and psychosis, immunosuppression, infections, hairy, myopathy, osteoporosis and avascular necrosis of bone, cataracts, and fat distribution, *et al*.

It has been demonstrated that as acute GVHD treatment response decreases, severity of the disease increases [84]. In a retrospective, 5-year survival in those patients response to steroid was significantly higher than those non-response to steroid (51% *versus* 32%). Similar result was also reported by Martin *et al*. [84, 85]. Unfortunately, only ∼60% of acute GVHD patients respond to systemic steroids and many of these responses are not durable [82, 86, 87]. The therapy effects of other agents in addition to prednisone on acute GVHD were studied for initial therapy [81, 88]. Agents evaluated in prospective studies have included Calcineurin inhibitors, MMF, pentostatin, etanercept,infliximab,Abs against IL-2R, horse ATG, anti-TNF drugs, however, most of these agents have proven futility [89–95].

### A recent phase II trial conducted by the Blood and Marrow Transplant

Clinical Trials Network (BMT-CTN) has identified the combination of corticosteroids and MMF as a promising strategy [94]. In a phase III study, which has been recently closed for accrual and the data analysis is being awaited (http://www.clinicaltrials.gov identifier NCT01002742), this combination was compared against standard corticosteroid therapy alone in the therapy of acute GVHD.

Non-glucocorticoid systemic immune suppressive agents for the first-line therapy of acute GVHD may be an alternative approach with comparable efficacy and less morbidity related to glucocorticoids [95], which requires further exploration.

### Second-line therapy

Acute GVHD is considered steroid-refractory when acute GVHD progresses within 3 days or is not improved after 5–7 days of initial treatment with 2 mg/kg dose methylprednisolone [82]. Sometimes, if acute GVHD is of a milder grade II, a longer observation interval of up to 2 weeks is acceptable [96]. Decisions to initiate secondary therapy should be made sooner for patients with more severe acute GVHD.

Very few prospective studies have evaluated the efficacy and safety of second-line therapy for acute GVHD, and interpretation of these studies is hampered by the lack of standardization. Agents that have been investigated over the last two decades in these trials include the following: low-dose MTX, MMF, extracorporeal photopheresis, IL-2R targeting, antibody therapy against CD3, CD7, CD25, CD52, CD147, IL-2R, IL-1, and TNF-α (*i.e*., basiliximab, daclizumab, denileukin, diftitox and alemtuzumab), horse ATG, etanercept, infliximab, and sirolimus, infusions of mesenchymal stem cells (MSCs) [27, 81, 97–116]. Intravenous immunoglobulin (IVIG) is effective, but with significant morbidity and mortality, mainly because of the infectious complications. Its cost and concern for impaired humoural recovery limit its widespread use [117–120]. More recently, numerous clinical trials using MSCs to treat acute GVHD have been reported [121–128]. Mesenchymal stem cells are suggested to suppress acute GVHD without impairing graft-versus-leukaemia effects and increasing systemic infections. However, there are many unsolved problems in the treatment of acute GVHD with MSCs (*e.g*., the source of MSCs, the single dose of MSCs, the total dose of MSCs and the interval of MSC administration). It is unclear whether MSCs preferentially suppress gut aGVHD or aGVHD in paediatric patients. Because few prospective comparative data on superior efficacy for any particular agent has been carried out, there are currently no criteria to identify patients who are likely to benefit from these second-line agents. The second-line regimen is chosen based on the effects of prior treatments, desired toxicity profile, considerations for drug interactions, logistical practicality, costs, and patient and physician preferences. Second-line treatments, especially those associated with the depression of T cells, are associated with increased infection and viral reactivation (including CMV, EBV, HHV-6, adenovirus, and polyoma [129, 130]. Many novel approaches are currently under investigation, but, to our knowledge to date, none of these approaches have achieved any improvements in overall survival in patients with steroid- refractory acute GVHD. Whether these approaches are truly representative of broader practices should be determined by retrospective studies on contemporary patients.

## Future directions

The mechanisms of acute GVHD have been progressively elucidated over recent years. Many approaches have been developed and are being under investigation to prevent and treat acute GVHD using experimental models, including IL-21 blockade, Histone deacetylase inhibitors inducible costimulator, CSF-1, glycogen synthase kinase 3 inhibotion, Human CD8+ Regulatory T Cells [131–137].

In recent years, plasma biomarkers have been identified and validated as promising diagnostic tools for acute GVHD and prognostic tools by the development of proteomics technology. These biomarkers (Albumin, CRP, CXCL10, HGF, L-2Rα, IL-6, IL-8, IL-10, IL-12, IL-18, KRT18 REG3α, TNF-α, *et al*.) may represent novel therapeutic targets that could be inhibited by future acute GVHD-specific drugs which have been reviewed recently by Paczesny [138]. Because these drugs would target the appropriate effector T cells, they should increase efficacy and lower toxicity. These biomarkers may facilitate timely and selective therapeutic intervention, but should be more widely validated and incorporated into a new grading system for risk stratification of patients and better-customized treatment.

Gene transfer technologies are also promising tools to manipulate donor T cell immunity to enforce graft-versus-tumour/graft-versus-infection while preventing or controlling acute GVHD. For this purpose, several cell and gene transfer approaches have been investigated at the pre-clinical level and implemented in clinical trials [139, 140].

Our group recently has been suggested and demonstrated the therapeutic effects of hydrogen on acute GVHD in a mice model [141, 142]. H_2_ exert anti-oxidative and anti-inflammatory effects with few toxic side effects. Mutagenicity, genotoxicity and subchronic oral toxicity of hydrogen in a rat model was assessed by Saitoh *et al*. [143]. Significant changes basophil ratio of blood in female rats and decreased aspartate aminotransferase and alanine aminotransferase in male rats were observed which were not considered biologically significant. Similar clinical chemistry parameters were also observed by Nakao *et al*. in human beings [144]. Because few side effects of H_2_ have been reported, it is a promising and novel finding, which is easy to be used in clinic. However, acute GVHD remain difficult to prevent and treat. The most effective approach to treat acute GVHD is likely to be one that disrupts all three phases of the acute GVHD cascade synergistically. In the future, we would like to see targeted interventions to prevent and treat acute GVHD.
